# Editorial

**Published:** 1869-03

**Authors:** 


### Editorial.

College Commencement.—The public Commencement of Chicago Medical College will take place in the College Hall, 1015 State Street, on the *fourth Tuesday* in March. There will be a public examination of the candidates for Graduation and the *reading* of Theses during the afternoon of the preceding day. The Commencement exercises proper will take place on the afternoon of Tuesday, and will consist in the distribution of Certificates to the undergraduates, the announcement of awards, the conferring of the Degrees, and a Valedictory Address by Prof. J. S. Jewell. Members of the profession, both in the city and the country, are invited to attend all these exercises.

Illinois State Medical Society.—We again remind our readers, that the next Annual Meeting of the Illinois State Medical Society will be held in Chicago, on the *third Tuesday* in May next. Drs. R. C. Hamill, S. Wickersham, Thomas Bevan, E. Powell, and G. C. Paoli, constitute the Committee of Arrangements.

Alumni Association of Chicago Medical College.—The second regular Annual Meeting of this Association will be held in the College, on Monday evening, twenty-second of March, at half-past 7 o'clock. An address will be delivered by the President of the Association, and matter of interest to all will be presented for consideration. Previous to which, it is earnestly to be hoped each *Alumnus* "will address a letter to the Secretary, giving a short history of his professional experience during the preceding year, and stating, at length, anything of special interest that may have come under his observation."

All Alumni of the Institution are earnestly invited to be present, to participate in, and contribute to, the interest of the meeting. S. A. McWilliams, *Sec'y*,
166 State St., Chicago.

### DELEGATES TO AMERICAN MEDICAL ASSOCIATION, ATTENTION.

The undersigned having been appointed a Committee to make arrangements for commutation of fare to and from the meeting of the Association, to be held in New Orleans, on the first Tuesday in May next, would have their work much facilitated if all those who wish to take the most direct, *time-saving* route, (which is by railroad,) will immediately inform the Committee of their wishes. We have ascertained that liberal commutation can be obtained from this city to New Orleans and return, provided a certain number of Delegates and Members will pledge themselves to go.

N. S. DAVIS,

R. C. HAMILL, } *Committee.*

A. FISHER,

NOTE.—The two principal routes of travel from this section of country to New Orleans are:—1st, All the way by Railroad, with only one *change*, which is at Louisville, and the journey accomplished in two and a half days, from Chicago to New Orleans. 2d, From Chicago to Cairo by Illinois Central Railroad, and thence by steamboat down the Mississippi to New Orleans, which cannot be safely estimated to consume less than *five* days.

The regular fare from Chicago to New Orleans by both these routes is $38.00. From information furnished by railroad agents here, and from a letter just received from Dr. Jas. F. Hibberd, of Richmond, Indiana, it is probable that arrangements can be made for commutation tickets over both these routes.

To complete the arrangements, it is very desirable that all the delegates and members of the Association who intend to go to New Orleans from the North-West, should immediately notify the Local Committee here, specifying the route they would prefer to go.

---

At a recent meeting of the Chicago Medical Society, the following form of a law was agreed to, and recommended to the

Legislature for adoption, by a nearly unanimous vote. It differs but little from the law now in force in Ohio, and is as follows:—

"AN ACT TO PROTECT THE PEOPLE OF ILLINOIS FROM EMPIRICISM AND IMPOSITION IN THE PRACTICE OF MEDICINE AND SURGERY.

"SECTION 1. *Be it enacted by the People of the State of Illinois, represented in General Assembly,* That it shall be unlawful for any person within the limits of said State, who has not attended at least two full courses of instruction, and graduated at some school of medicine, either of the United States or of some foreign country, or who cannot produce a certificate of qualification from some State, district, or county medical society, composed of not less than twelve active members, and is not a person of good moral character, to practise medicine in any of its departments for reward or compensation, or attempt to practise medicine, or prescribe medicine or medicines, for reward or compensation, for any sick person within the said State of Illinois.

"§ 2. Any person living in the State of Illinois, or any person coming into said State, who shall practise medicine, or attempt to practise medicine, in any of its departments, or perform, or attempt to perform, any surgical operation upon any person within the limits of said State, in violation of section one of this act, shall, upon conviction thereof, be fined not less than fifty, nor more than one hundred, dollars for such offence; and, upon conviction for a second violation of this act, shall, in addition to the above fine, be imprisoned in the county jail of the county in which such offence shall have been committed for the term of thirty days. And in no case wherein this act shall have been violated, shall any person so violating receive a compensation for services rendered: *Provided,* Nothing herein contained shall in any way be construed to apply to any person practising dentistry.

"§ 3. This act shall take effect and be in force on and after the first day of September, 1869."

Individually, we do not anticipate much benefit from legislation of any kind concerning the practice of medicine. If we are to have any law, however, we should much prefer this simple enactment to any other that has been proposed; unless we could have a thoroughly competent Board of Examiners for the State, selected in some way that would secure the freedom of its members from partisanship, and by whom all persons proposing to practise medicine and surgery in this State should be thoroughly examined, whether they be graduates of some medical school or not.

---

THE HEALTH-LIFT—ITS THEORY AND PRACTICE.—This is the title of a neatly-printed and bound monograph, written, we presume, by Frank W. Reilly, M.D., who has charge of a Health-Lift establishment in this city. By Health-Lift here, is meant a regular daily exercise at lifting, for the purpose of developing the strength and health of the physical system. As practised under the direction of Dr. Reilly, it is a decided improvement over the ordinary gymnastic exercises. Regular habitual muscular exercise, as a means of preserving and restoring health, should receive far more attention than has hitherto been given to it.

---

ERRORS.—In the February number of the EXAMINER, Dr. Curtis relates an unusual case of retention or suppression of urine.

In setting up the article, the printers omitted a few words, which made it read, "20 grs. of calomel every three hours." It should have been, a "powder of calomel 4 grs., jalap 6 grs., and cream tartar 20 grs."

On page 89 of the same number of the EXAMINER, is a prescription which should have been printed ounces instead of drachms.

---

A REBELLIOUS CASE OF APHONIA, instantly cured by electrical excitation of the inferior laryngeal nerve, has been communicated by Dr. R. Philipeaux to the *Gazette Medicale de Lyon*, 1868, No. 30.—*Medical Record.*

---

THE CHAIR OF OBSTETRICS AND MED. JURISPRUDENCE IN THE MASS. MED. COLLEGE FILLED.—Dr. Chas. E. Buckingham, formerly Adjunct Professor of Theory and Practice, has been appointed by the Corporation to the vacant chair of Obstetrics and Med. Jurisprudence in the Massachusetts Medical College. —*Ibid.*

The Next Meeting of the American Medical Association.—We hope that the approaching meeting of the American Medical Association will be fully attended by members of the profession from the Northern States. The season of the year, the convenience of access to New Orleans by steamboat, and the well-known delightful climate of the lower Mississippi in the spring, are inducements which should alone decide many a hard-worked doctor to take the trip, "and so to interpose a little ease."

The expensiveness of the journey will indeed deter many, and this difficulty, therefore, should be reduced as much as possible. A steamboat could undoubtedly be chartered at Cincinnati or Louisville for the round trip, at a very much less rate per person than for each to go by the usual conveyances. While at New Orleans those who wished could take their meals and sleep on board.

All those who are desirous of entering into this arrangement should send their names and address, and the names of the members of their families they might wish to accompany them, to Dr. Wm. B. Atkinson, Permanent Secretary, No. 1400 Pine Street, Philadelphia, Pa., without delay. Let as many as can, conclude to go, as in this case it is not only the more the merrier, but also the more the better fare and the lower price.

It is understood that unless enough names are received to make this method of taking the journey the cheapest, it will not be adopted.—*Medical and Surgical Reporter.*

---

The Fungus Theory of Disease Doubtful.—In a short communication to the *Centralblatt*, Drs. Bergmann and Schmiedeberg describe a crystalline substance, to which they have applied the name "Sulphate of Sepsin," obtained from putrefying materials, and which they believe represents the proper poison of organic substances undergoing this kind of fermentation. It is obtained by diffusion through parchment-paper, precipitation with corrosive sublimate from an alkaline solution, removal of the mercury by silver, of silver by sulphuretted hydrogen, evaporation, and purification of the residue. Large well-defined, acicular needles are thus obtained, which are deliquescent in the air, and, exposed to heat, melt and carbonize. They possess a powerfully poisonous action. A solution containing scarcely more than one-hundredth of a gramme was injected into the veins of two dogs. Vomiting was immediately induced, and after a short time diarrhœa, which in the course of an hour became bloody. After nine hours the animals were killed, and on examination, their stomachs and large intestines were found ecchymosed, and the small intestine congested. Frogs could be killed in the same manner.—*Lancet, Oct. 17, 1868, p. 518.*

---

A SUCCESSFUL OPERATION FOR THE TRANSFUSION OF BLOOD was recently performed by Dr. Enrico Albanese at the hospital of Palermo, Sicily. A youth aged seventeen, named Giuseppe Ginazzo, of Cinisi, was received at that establishment on the 29th of September last, with an extensive ulceration of the leg, which in the end rendered amputation necessary, the patient being very much emaciated, and laboring under fever. The operation reduced him to a worse state than ever, and it became apparent that he was fast sinking; the pulse being imperceptible, the eyes dull, and the body cold. In this emergency, Dr. Albanese had recourse to the transfusion of blood as the only remedy that had not yet been tried. Two assistants of the hospital offered to have their veins opened for the purpose, and thus at two different intervals 220 grammes of blood were introduced into the patient's system. After the first time he recovered the faculty of speech, and stated that before he could neither see nor hear, but felt as if he were flying in the air. He is now in a fair state of recovery.—*Ibid.*

---

A NEW URETHROSCOPE—and we like the name better than *endoscope*—has been devised by Dr. Langlebert, of Vichy, which can readily be used by daylight, or by the illumination of an ordinary lamp or candle. It is very simple in construction. A full description, with woodcut, appears in *La France Medicale*, 1868, No. 77.—*Ibid.*

---

ROYAL INSTITUTION.—Dr. Odling, Prof. Chem. at St. Bartholomew's Hospital, has been elected Fullerian Prof. Chem. in this inst. to succeed the illustrious Faraday.

---

The Academy of Sciences of Paris has received the sum of 60,000 francs, the interest of which will every third year be offered as a prize for the best essay on embryology. M. Serres was the judicious owner.—*Medical and Surgical Reporter.*

---

OVARIOTOMY.—Dr. Dunlop, of Springfield, Ohio, since 1843, has performed ovariotomy on 38 patients. Only two were without anæsthetics; and all were by the long incision. Thirteen of his patients were unmarried. Nine died after the operation. Three of the successful cases died of other diseases afterwards; the rest are all now living, and in good health.—*N. Y. Med. Record.*

---

To Prevent the Coagulation of the Blood during Transfusion.—Dr. J. Braxton Hicks (*British Medical Journal*) accomplishes this by a solution of phosphate of soda mixed with the blood as it flows from the supplier. The greatest danger in the operation is thus avoided, whilst the solution is in no degree incompatible with the integrity or normal qualities of the blood. —*Pacific Medical and Surgical Journal.*

---

Solvent for Gallstone and Cholesterine.—Dr. Buckler (*Am. Jour. of Med. Science*) recommends the following prescription in the cholesterine diathesis:—℞. Hydrat. succin. of iron, ℥iss.; water, ℥iss. Dose, a teaspoonful after each meal, to be taken six months, if necessary.—*Medical Record.*

---

There are so many quacks in London who assume the title of M.D., that the publishers of the London Directory have determined to expunge in future editions of that work, from the list comprising the names of physicians and surgeons, those not registered under the Medical Act of 1858. If our publishers of directories would dare to follow such an example, what a small list of real doctors there would be!—*Medical Record.*

---

Antidote for Carbolic Acid.—Next to the stomach-pump, in poisoning with this acid, the best antidote is large doses of olive or almond oil, with a little castor oil. Oil is a solvent, and therefore a diluent of carbolic acid, and may be used to stop the corrosive effect of the acid, when its action on the skin is too violent.—*Journal of Cutaneous Medicine.*

---

Pensions to Widows of Physicians.—The Italian Chamber has passed an act granting to the widows of physicians who fell victims to their calling during cholera epidemics, a pension of from 100 to 200 dollars a year, according to the number of children.—*N. Y. Med. Record.*

---

Best Book for Everybody.—The new illustrated edition of Webster's Dictionary, containing three thousand engravings, is the *best book for everybody* that the press has produced in the present century; and should be regarded as indispensable to the well-regulated home, reading-room, library, and place of business.—*Golden Era.*

MORTALITY FOR THE MONTH OF DECEMBER, 1868:—

| | |
|---|---|
| Accident, drowned | 2 |
| " burned, | 1 |
| " overdose of morphine, | 1 |
| " jumping fm. window, | 2 |
| " run over by " wagon, | 2 |
| " R. R. cars, | 1 |
| " scalding | 2 |
| Abdominal aorta, aneurism of, | 1 |
| Angina, | 2 |
| Apoplexy, | 3 |
| Aphthæ, | 1 |
| Atrophy, | 1 |
| Atelectasis pulmonium | 1 |
| Asphyxia, | 1 |
| Biliary calculi, | 1 |
| Births, premature, | 7 |
| " still | 37 |
| Brain, congestion of, | 6 |
| " disease of, | 1 |
| " effusion into vertricles, | 1 |
| " inflammation, | 5 |
| " softening of, | 1 |
| " " and old age, | 1 |
| Bronchitis, | 5 |
| " capillary, | 4 |
| Carditis rheumatic, | 1 |
| Chord, umbilical injury of, | 1 |
| Cholera-infantum, | 1 |
| " morbus, | 1 |
| Childbirth | 1 |
| Convulsions, | 40 |
| Croup, | 10 |
| " membranous, | 3 |
| Debility, general, | 2 |
| Diarrhœa, | 2 |
| " chronic, | 2 |
| Diphtheria, | 6 |
| " and angina | 1 |
| " and paralysis, | 1 |
| " pneumonia | 1 |
| Disease, congenital, cerebral, and spinal, | 1 |
| Dropsy, | 5 |
| " ovarian, | 1 |
| Dysentery | 2 |
| Enteritis, | 1 |
| Enterocolitis | 1 |
| Encephatitis, | 1 |
| Epilepsy | 1 |
| Exposure and neglect, | 1 |
| Fever, puerperal, | 4 |
| " scarlet, | 31 |
| " " malignant, | 2 |
| " " & angina, | 1 |
| " " & convulsions | 1 |
| " " & laryngitis, | 1 |
| " " & nephritis, | 1 |
| " typhoid, | 19 |
| Gastritis | 1 |
| Hæmatemesis, | 1 |
| Hepatitis, | 1 |
| " chronic and excitis, | 1 |
| " " and dropsy, | 1 |
| Heart disease of, | 4 |
| " " and dropsy | 1 |
| " dropsy of, | 1 |
| " congestion of, and phthisis pulmonalis, | 1 |
| " organic disease of | 1 |
| " hypertrophy of, | 1 |
| " valvular disease | 1 |
| Hydrocephalus, | 5 |
| " acute, | 1 |
| Hydropericarditis, | 1 |
| Hydrothorax, | 1 |
| Inanition, | 8 |
| Intemperance, and exposure, | 3 |
| Liver, cirrhosis of, | 1 |
| " organic disease, | 1 |
| Lithotomy and urinary infiltration, | 1 |
| Lungs, congestion of, | 4 |
| Measles, | 3 |
| Meningitis, | 6 |
| " acute, | 1 |
| " cerebro-spinal, | 3 |
| Meningitis tubercular, | 1 |
| Mouth, canker sore | 1 |
| Metro-peritonitis, | 1 |
| Miscarriage, | 1 |
| Nephritis, acute, | 1 |
| Old age, | 5 |
| Œdema, pulmonium, and diphtheria, | 1 |
| Paralysis, | 1 |
| Parotitis, and encephatitis, | 1 |
| Peritonitis, | 5 |
| Peritonæum, cancer of | 1 |
| Phthisis pulmonalis, | 21 |
| " and pneumonia | 1 |
| Pneumonia, | 31 |
| " broncho, | 2 |
| " typhoid, | 7 |
| " and diphtheria, | 1 |
| " and old age | 1 |
| Phlebites, uterine, and erysipelas, | 1 |
| Rectum, cancer of, | 1 |
| Skull, fracture of, | 1 |
| Stomach, cancer of, | 2 |
| " and bowels, inflammation of, | 1 |
| Suicide by pistol shot, | 1 |
| " hanging, | 1 |
| " morphine, " from insanity, | 1 |
| Small-pox, | 2 |
| Tabes mesenterica, | 11 |
| Tetanus, | 2 |
| " and convulsions | 1 |
| Teething, | 4 |
| Throat, ulcerated sore, | 1 |
| Uterus, hemorrhage of, | 1 |
| " cancer of, | 1 |
| Unknown, | 1 |
| Vitality, deficient, | 3 |
| Varicella, | 1 |
| Whooping-cough, | 6 |
| " " and pneumonia, | 1 |
| Total, | 374 |

AGES.

| | | | | | |
|---|---|---|---|---|---|
| Under 1 | 109 | 20 to 30 | 27 | 70 to 80 | 8 |
| 1 to 3 | 71 | 30 to 40 | 46 | 80 to 90 | 1 |
| 3 to 5 | 23 | 40 to 50 | 23 | Unknown, | 2 |
| 5 to 10 | 26 | 50 to 60 | 10 | | |
| 10 to 20 | 14 | 60 to 70 | 14 | Total | 374 |

### COMPARISON.

| | | |
|---|---|---|
| Deaths in Dec., 1868, -- 374 | Deaths in Dec., 1867, --409 | Decrease, -- 25 |
| Deaths in Nov., 1868, -------- 401 | Decrease, ---------------------- 27 | |
| Males, ---------- 203 | Females, --------- 171 | Total, ---------- 374 |
| Single, ---------- 277 | Married, --------- 97 | Total, ---------- 374 |
| White, ---------- 368 | Colored, ----------- 6 | Total, ---------- 374 |

### NATIVITY.

| | | | | | |
|---|---|---|---|---|---|
| Bavaria, ---------- | 2 | England, ---------- | 11 | Poland, ---------- | 1 |
| Bohemia, ---------- | 7 | France, ---------- | 1 | Scotland, ---------- | 5 |
| Canada, ---------- | 5 | Germany, ---------- | 39 | Sweden, ---------- | 8 |
| Native Chicago, ---- | 71 | Holland, ---------- | 3 | Unknown, ---------- | 2 |
| Foreign " ---- | 127 | Ireland, ---------- | 46 | | |
| U. S., other parts, --- | 42 | Norway, ---------- | 4 | Total, ---------- | 374 |

### MORTALITY BY WARDS FOR THE MONTH.

| Ward. | Mortality. | Pop. in 1868. | One death in | Ward. | Mortality. | Pop. in 1868. | One death in |
|---|---|---|---|---|---|---|---|
| 1--- | 7 | 9,094 | 1,299 | 14--- | 20 | 14,839 | 742 |
| 2--- | 15 | 13,074 | 871 | 15--- | 31 | 21,078 | 680 |
| 3--- | 20 | 15,076 | 753¾ | 16--- | 12 | 15,465 | 1,288¾ |
| 4--- | 20 | 17,796 | 889¾ | County hosp. | 7 | | |
| 5--- | 22 | 16,033 | 728¾ | Chi. River, | 1 | | |
| 6--- | 25 | 13,083 | 523⅓ | Mercy Hosp. | 2 | | |
| 7--- | 56 | 25,492 | 455 1-5 | St.Luke's H's. | 3 | | |
| 8--- | 28 | 15,813 | 564¾ | Immigrants | 6 | | |
| 9--- | 15 | 19,297 | 1,286½ | Marine Asyl. | 2 | St.Jo. Orph. Asyl. | 1 |
| 10--- | 13 | 12,925 | 840½ | Soldiers Home | 1 | Prot. Orph. Asylum | 2 |
| 11--- | 20 | 14,340 | 717 | L. Michigan, | 1 | Home for Friendless, | 7 |
| 12--- | 18 | 17,485 | 971½ | | | | |
| 13--- | 19 | 11,164 | 587½ | | | | |

Total, ---------------------------------- 374

### MORTALITY FOR THE MONTH OF JANUARY, 1869:—

| | |
|---|---|
| Accidents, ---------- | 6 |
| Abscess, lumbar, ---- | 1 |
| Anæmia, ---------- | 1 |
| Angina, ---------- | 1 |
| Apoplexy, ---------- | 3 |
| " and splinetis | 1 |
| Aphthæ ---------- | 1 |
| Ascites, ---------- | 2 |
| Atelectasis pulmonium | 1 |
| Bowels, inflammation, | 2 |
| " cancer of, ---- | 1 |
| Brain, congestion of,- | 3 |
| " " " & scarlet fever, | 2 |
| " compression of, | 1 |
| " inflammation, - | 5 |
| Bronchitis, ---------- | 3 |
| " & apoplexy | 1 |
| " & emphysema, ---- | 1 |
| " & phthisis pulmonalis, | 1 |
| " & capillary | 3 |
| Cancer, ---------- | 2 |
| Cerebritis, ---------- | 1 |
| Cholera infantum, ---- | 1 |
| Convulsions, -------- | 49 |
| Croup, ---------- | 5 |
| " membranous, -- | 5 |
| " spasmodic, ---- | 1 |
| " and diptheria, - | 1 |
| Cyanosis, ---------- | 1 |
| Cyanche trachealis, -- | 1 |
| Debility, general, ---- | 3 |
| Diabetes, ---------- | 1 |
| Diarrhœa, ---------- | 2 |
| " and indigestion, ----- | 1 |
| Diptheria, ---------- | 2 |
| Dropsy, ---------- | 3 |
| " and scarlet fever, -------- | 2 |
| Dropsy of abdomen, -- | 1 |
| Dysentery, ---------- | 1 |
| Endocarditis and convulsions, ---------- | 1 |
| Enteritis, ---------- | 2 |
| Epilepsy, by worms -- | 1 |
| Erysipelas, ---------- | 3 |
| " phlegmonous, ---- | 1 |
| " and pyæmia | 1 |
| Exhaustion, nervous, - | 1 |
| Fever, puerperal, ---- | 4 |
| " scarlet, ------- | 40 |
| " " and meningitis, ----- | 2 |
| " " malignant, | 2 |
| " typhoid, ------ | 5 |
| " " and chronic dysentery, | 1 |
| Gangrene, dry, ------ | 1 |
| Gastritis, ---------- | 1 |

| | |
|---|---|
| Gastritis and phthisis pulmonalis, | 1 |
| Hæmoptysis, | 1 |
| Heart, fatty degeneration, of, | 1 |
| " disease of, | 2 |
| " organic disease, | 1 |
| " valvular " | 2 |
| Hepatitis, chronic, | 1 |
| Hernia, strangulated | 1 |
| Hydrophobia, | 1 |
| Hydrocephalus, | 6 |
| " acute, | 2 |
| Icterus, | 1 |
| Inanition, | 8 |
| Inflammat'n, peritoneal | 1 |
| Intemperance, | 1 |
| " and exposure | 1 |
| " and rheumatism, | 1 |
| Larynx, ulceration of, | 1 |
| Laryngitis, | 1 |
| Liver, congestion of, | 1 |
| " disease of, and intemperance, | 1 |
| " induration of, | 1 |
| Lungs, congestion of, | 1 |
| Lungs, paralysis and congestion of, | 1 |
| " hemorrhage of, and phthisis pulmonalis, | 1 |
| Manslaughter, | 1 |
| Measles and diptheria | 1 |
| Metro-peritonitis, | 1 |
| Meningitis, | 5 |
| " acute | 1 |
| " cerebro-spinal | 6 |
| " tubercular, | 3 |
| Old age, | 8 |
| Œdema, pulmonium, | 2 |
| Parotid glands, infiltration of, | 1 |
| Paralysis, | 2 |
| Pericarditis | 1 |
| Peritonitis, | 2 |
| Phthis pulmonalis, | 45 |
| Pleurisy, and convulsions, | 1 |
| Pneumonia, | 27 |
| " and bronchitis, | 2 |
| " and convulsions, | 1 |
| " broncho, | 1 |
| " typhoid, | 2 |
| Prolapsus funis, | 1 |
| Scrofula, | 1 |
| " necrosis of, and diarrhœa, | 1 |
| Suicide, | 1 |
| Small-pox | 1 |
| Spina bifida, | 1 |
| Stomach, cancer of, | 3 |
| Tabes mesenterica, | 8 |
| Teething, | 3 |
| Throat, putrid sore, | 1 |
| Tongue, cancer of, | 1 |
| Tumor, abdominal, | 1 |
| Uterus, cancer of, | 2 |
| " hemorrhage of, | 1 |
| Varicella, and pneumonia, | 1 |
| Whooping-cough, | 7 |
| " and bronchitis, | 3 |
| " and pneumonia, | 1 |
| Unknown, | 2 |
| Total, | 382 |

AGES.

| | |
|---|---|
| Under 1 | 123 |
| 1 to 3 | 69 |
| 3 to 5 | 28 |
| 5 to 10 | 23 |
| 10 to 20 | 18 |
| 20 to 30 | 19 |
| 30 to 40 | 36 |
| 40 to 50 | 28 |
| 50 to 60 | 16 |
| 60 to 70 | 7 |
| 70 to 80 | 11 |
| 80 to 90 | 3 |
| 117 | 1 |
| Total, | 382 |

NATIVITIES.

| | |
|---|---|
| Atlantic Ocean, | 1 |
| Bohemia, | 4 |
| Canada, | 1 |
| Chicago, Native, | 81 |
| Chicago, Foreign, | 121 |
| U. S., other parts, | 57 |
| England, | 13 |
| France, | 2 |
| Germany, | 44 |
| Holland, | 1 |
| Ireland, | 36 |
| Italy, | 2 |
| Norway, | 9 |
| Scotland, | 2 |
| Sweden, | 7 |
| Unknown, | 1 |
| Total, | 382 |

MORTALITY BY WARDS FOR THE MONTH.

| Ward. | Mortality. | Pop. in 1868. | One death in |
|---|---|---|---|
| 1 | 3 | 9,094 | 3,031 |
| 2 | 21 | 13,074 | 622 |
| 3 | 17 | 15,076 | 887 |
| 4 | 15 | 17,796 | 1,186 |
| 5 | 26 | 16,033 | 617 |
| 6 | 16 | 13,083 | 818 |
| 7 | 34 | 25,492 | 750 |
| 8 | 24 | 15,813 | 659 |
| 9 | 28 | 19,297 | 689 |
| 10 | 11 | 12,925 | 1,175 |
| 11 | 23 | 14,340 | 623 |
| 12 | 30 | 17,485 | 583 |
| 13 | 20 | 11,164 | 558 |
| 14 | 21 | 14,839 | 707 |
| 15 | 34 | 21,078 | 620 |
| 16 | 34 | 15,465 | 455 |
| County Hosp. | 12 | | |
| Marine Hosp. | 1 | | |
| H'me for Fr'ndless, | 4 | | |
| Prot. Orphan Asylum, | 1 | | |
| Total, | 382 | | |

COMPARISON.

| | | |
|---|---|---|
| Deaths in Jan., 1869, ....382 | Deaths in Jan., 1868, ....438 | Decrease, .... 56 |
| Deaths in Dec., 1868, ............374 | Increase, ........................ 8 | |
| Males, ............208 | Females, ............174 | Total, ..........382 |
| Single, ............279 | Married ............103 | Total, ..........382 |
| White, ............380 | Colored, ............ 2 | Total, ..........382 |

An Aged Primipara.—Dr. Cachot, of St. Mary's Hospital, London, recently delivered in that institution a female of her first child, at the age of 53 years, and again in sixteen months. In both confinements the labor was tedious, from inertia of the uterus, and forceps were required. The mammary glands produced no milk, but were enlarged. The children lived in both cases.—*Ibid.*

